# The Interaction between Arbuscular Mycorrhizal Fungi (AMF) and Grass Endophyte (*Epichloë*) on Host Plants: A Review

**DOI:** 10.3390/jof10030174

**Published:** 2024-02-26

**Authors:** Youlei Shen, Tingyu Duan

**Affiliations:** 1 State Key Laboratory of Herbage Improvement and Grassland Agro-Ecosystems, Lanzhou University, Lanzhou 730020, China; 220220902331@lzu.edu.cn; 2 College of Pastoral Agriculture Science and Technology, Lanzhou University, Lanzhou 730020, China; 3 Key Laboratory of Grassland Livestock Industry Innovation, Ministry of Agriculture and Rural Affairs, Lanzhou 730020, China; 4 Engineering Research Center of Grassland Industry, Ministry of Education, Lanzhou 730020, China; 5 Gansu Tech Innovation Center of Western China Grassland Industry, Lanzhou 730020, China

**Keywords:** arbuscular mycorrhizal fungi, grass endophyte, symbiosis, co-colonization, interaction, stress tolerance

## Abstract

In nature, plants frequently experience concurrent colonization with arbuscular mycorrhizal fungi (AMF) and grass endophytes (*Epichloë*). These two fungi assist in mineral uptake and stress tolerance by the host. Despite the abundance of recent studies exploring the individual functions of these fungi in diverse ecosystems, research on the effects of the interaction between these two symbiotic fungi on the host, particularly in agricultural production and ecological conservation. This review provides an overview of the current knowledge regarding the interaction between AMF and grass endophytes and their synergistic effects on host plants in response to abiotic and biotic stress, while also outlining prospects for future research in this field. This knowledge not only enhances our comprehension of complex interaction effects between the two fungi, but also facilitates the optimal utilization of fungal resources, contributing to ecological construction and higher agricultural production.

## 1. Introduction

Natural ecosystems comprise numerous microbial communities. Plants can form symbiotic associations with diverse fungi [1,2,3]. Cold-season grasses in grasslands can establish symbiotic relationships with arbuscular mycorrhizal fungi (AMF) of the phylum Glomeromycota and grass endophytes of the genus *Epichloë* [4,5,6].

Arbuscular mycorrhizal fungi constitute diverse and significant microorganisms in soils and various ecosystems, even those in adverse conditions [7,8,9,10]. Over 80% of terrestrial plants roots form symbiotic relationships with AMF [11]. AMF increases the root absorption surface area, thereby enhancing the uptake of nutrients such as nitrogen and phosphorus, promoting photosynthesis, and regulating photosynthetic product distribution, ultimately improving plant growth [12,13,14,15]. Furthermore, AMF promotes the host plant’s resistance to adverse environmental stressors, including drought, heavy metals, microplastics, and biotic stresses such as pathogens and herbivory insects [16,17,18,19,20,21,22,23,24,25]. In reciprocation, plants supply AMF with the necessary carbon sources for their growth and survival [26,27]. The enduring relationship between AMF and host plants, established over an extended period of evolution, is characterized as a well-established symbiotic association [15,28,29].

Grass endophytes are fungi of the genus *Epichloë* that form symbiotic relationships strictly with certain species of C_3_ grasses in the Pooideae subfamily, including numerous forage and turf grasses [30]. The endophytic colonization by the grass endophyte occurs within the intercellular spaces of the host’s sheaths and leaves and is passed to the host’s progeny through vertical transmission, seed dispersal, or both [31]. The host plant nourishes and creates a conducive environment for the endophyte’s growth. In return, the endophyte enhances the host’s resilience to both biotic and abiotic stressors, improving the nutrient absorption efficiency of the host plant [32,33,34,35]. Most cold-season grasses can establish symbiotic relationships with endophytic fungi belonging to the genus *Epichloë*.

AMF and grass endophytes are important below-ground and above-ground microorganisms that often colonize the same host plants, forming complex AMF-*Epichloë*-plant associations called tripartite interactions. Understanding how plant-microbiome and microbe-microbe interactions occur has been the subject of increasing research interest [36,37,38,39,40]; particularly, the symbiosis patterns of multiple microorganisms, including both above-ground and below-ground microorganisms, and the ecological mechanisms underlying host plant-microbial community interactions. Such research can provide valuable insights for future microbial and genetic engineering. Knowledge of the interaction between AMF, grass endophytes, and their host plants in ecosystems remains scanty. To shed light on this topic, we present a review of recent articles on the interactions between AMF and grass endophytes. Our aims were to (1) provide an overview of how grass endophytes and AMF promote stress tolerance in host plants; (2) describe the mechanism of interaction between these two symbionts with host plants; and (3) identify priority areas for future research on the tripartite interaction between AMF, grass endophytes, and host plants.

## 2. Effect of Grass Endophyte on Arbuscular Mycorrhiza

### 2.1. Effect of Grass Endophyte on Mycorrhizal Colonization

Grasses are usually simultaneously colonized by grass endophytes and AMF, and these two symbiotic fungi inhabit both the above-ground and below-ground parts of the host plant [30,41]. Numerous studies have investigated how grass endophytes affect the rate of mycorrhizal colonization. The specific outcome is contingent upon the grass species and the particular combination of grass endophyte and mycorrhizal fungi involved, as detailed in Table 1. The main grass species studied in regard to the above association include *Lolium perenne*, *Lolium multiflorum*, *Achnatherum sibiricum*, *Schedonorus arundinaceus*, *Bromus auleticus*, *Leymus chinensis*, *Festuca paniculata*, *Elymus hystrix*, and *Poa bonariensis* (Lam.) *Kunth*.

The presence of grass endophytes can cause several effects on mycorrhizal colonization rates, ranging from negative [42,43] to positive [44], or neutral [45]. An initial investigation into the effect of grass endophytes on mycorrhizal colonization by Chuchou et al. [42] revealed that the concurrent infection of *Festuca arundinacea* with *Epichloë coenophiala* and *Funneliformis mosseae* decreased the root colonization rates by 41.2%. Other researchers have similarly found that infection of *Lolium perenne* with *Epichloë festucae* var. *lolii* reduced colonization by mycorrhizal *Claroideoglomus etunicatum* [43]. However, the opposite outcome was observed when host plants were exposed to stress. For example, *Epichloë* endophytes increased mycorrhizal colonization by 85.21% when *Lolium perenne* was infected with the pathogenic fungi *Bipolaris sorokiniana* [44]. Nevertheless, numerous studies indicate that grass endophytes do not affect mycorrhizal colonization [45]. Thus, we assumed that the effect of grass endophytes on mycorrhizal colonization rates depends on specific interaction outcomes among the mycorrhizal fungi, grass endophytes, host plant species, and environmental conditions. Further studies on the interactions between different AMF and grass endophytes, and the underlying mechanisms, are needed.

A few studies have investigated how mycorrhizal fungi affect the effect of grass endophytes on host plants [46,47,48]. For instance, Liu et al. (2011) found a negative correlation between the concentration of grass endophyte and mycorrhizal fungi colonization rate. The reduction in mycorrhizal fungi colonization positively correlated with soil P content and was dependent on the ryegrass cultivar and the grass endophyte strains [46]. Mack and Rudgers (2007) found that AMF had no effect on grass endophyte density [47], while Liu et al. (2020) found AMF could have a positive, negative, or no impact on grass endophyte concentration; the increased impact mostly occurred with *Glomus intraradices* individuals and its mix with other AMF [48]. Liu et al. (2011) further found that the inhibition of AMF by grass endophytes is linked with the reduction of alkaloids in the leaf blade and pseudostems of perennial ryegrass by Glomus [46]. More research is required to clarify the mutual benefits of the symbiotic association between grass endophytes and AMF colonization and the function of alkaloids, plant defenses, and signal molecules in these associations.

**Table 1:** Effect of grass endophytes on the mycorrhizal root colonization of host plants.

Host Plants	Grass Endophyte	AM Fungi	Study Site	Influence	Mechanism	References
*Agrostis capillaris*	*Epichloë* sp.	Live soil inoculation	Field	No impact.	/	[45]
*Bromus auleticus*	*Epichloë pampeana; Epichloë tembladerae*	Live soil inoculation	Field	+18% at 6 months after fertilization.	Genotype of host plants, and the exudate of different profile of compounds.	[49]
*Bromus auleticus*	*Epichloë* sp.	Living vertisol soil	Field	+21%~33%, +29% of abuscules and vesicles of the other neighbor grasses.	Grass-*Epichloë* association generate a soil environment through secreting root exudates.	[5]
*Bromus auleticus*	*Epichloë pampeana*	Living field soil	Greenhouse	+19%~43%.	The soil types of (agriculture soil and non-agriculture soil).	[50]
*Elymus hystrix*	*Epichloë elymi*	*Glomus claroideum*, *Glomus mosseae*	Greenhouse	−13%~19% when inoculated with *G. claroideum* but +15% with the *G. mosseae*.	Identity of the AMF species.	[51]
*Festuca arundinacea Schreb.*	*Acremonium coenophiulum* (*Epichloë coenophialum*)	*Funneliformis mosseae*	Greenhouse	−41.2%.	The toxic metabolites transferred to the root.	[42]
*Hordeum comosum*	*Epichloë tembladerae*	Living grassland soil	Field	+8%.	Differentiation of the plant niche and the external precipitation.	[52]
*Leymus chinensis*	*Epichloë bromicola*	Living grassland soil	Field	+15%.	Endophyte affected the soil properties.	[53]
*Lolium arundinaceum*	*Epichloë coenophialum*	*Funneliformis mosseae*; *Claroideoglomus etunicatum*	Greenhouse	−1.1% in saline-alkali stress of colonization rate of *F. mosseae*; +30.7%~38% colonization rate of *C. etunicatum*; +8%~32.2% colonization rate of the mixture of FM and CE.	Species of AMF and environmental conditions.	[54]
*Lolium perenne* (Fenneama and AberDart)	*Neotyphodium lolii* *(Epichloë festucae* var. *lolii*)	*Glomus intraradices*; *Glomus mosseae*	Greenhouse	−72.7% of Fenneama varieties, but had no effect on AberDart.	Species and specific of AMF and host plant.	[46]
*Lolium perenne*	*Epichloë festucae* var. *lolii*	*Claroideoglomus etunicatum*	Greenhouse	Much lower AMF colonization rate at 70% soil water contents.	Soil water contents.	[55]
*Schedonorus arundinaceus*	*Epichloë coenophiala*	Living pasture soil	Field	−53.6%.	Competition of C of *Epichloë* and AMF from host plant.	[56]
*Schedonorus phoenix*	*Neotyphodium coenophialum* (*Epichloë coenophialum*)	live soil inoculum	Greenhouse	−50.2%.	/	[47]

### 2.2. Mechanisms Underlying the Effect of Grass Endophyte on Mycorrhizal Colonization

The mechanism through which *Epichloë* endophytes affect the colonization pattern of AM fungi in the roots is not well understood. As mentioned in the preceding section, environmental conditions, external stresses (biotic or abiotic stresses), host plant species, and AMF species, among other factors, affect the colonization of arbuscular mycorrhizal fungi [57,58,59,60]. Hence, the mechanisms by which root-symbiont interactions are affected by *Epichloë* are very complex and require an in-depth investigation.

The effect of grass endophytes on the colonization of AM fungi can be categorized into direct and indirect modes. Compared to AMF, *Epichloë* endophytes have better spatial advantages. *Epichloë* is seed-borne and mainly exists in the above-ground stems and leaves of plants [30]. When AMF and grass endophytes concurrently colonize a host plant, both fungi rely on plant-derived carbon sources for growth. However, due to their time and spatial advantage within the above-ground stems and leaves, endophytes are granted preferential access to photosynthetic carbohydrates, potentially diminishing the availability of these products for AMF utilization. This resource competition may consequently inhibit the infection of AM fungi [47].

In AMF-grass endophyte-plants systems, *Epichloë* increases shoot/root phosphorus concentrations and net photosynthesis rates [44,52,55,61,62]. In this association, AMF provides a substantial amount of phosphorus-containing compounds to host plants in exchange for photosynthetic products [63,64]. In soil nutrient deficiency cases, the host’s reliance on AM fungi may decrease. Moreover, it has been observed that the rhizosphere of plants colonized with grass endophytes exhibits higher aggregate stability compared with those not colonized [65]. Thereby, we hypothesize that the variations in plant growth and nutrient availability resulting from colonization with grass endophytes can indirectly impact the diversity of the microbial community, altering mycorrhizal colonization [66].

Compounds exuded from the roots of plants greatly promote root mycorrhizal colonization, and identifying those compounds is essential for unraveling the mechanisms of root mycorrhizal colonization. Grass endophytes protect their host plants by producing secondary metabolites, including Loline, Peramine, Ergot, and Lolitrems [67], which can deter herbivore and insect feeding, providing direct plant defense [68]. In the majority of studies, the alkaloids produced by grass endophyte have been detected in the above-ground tissues of host plants, such as shoots, leaves, and even seeds [30]. One study revealed that alkaloids are also present in the soil [69]. An in vitro experiment conducted on the native grass *Bromus setifolius* revealed that exudates of *Epichloë* species and endophyte-infected plants improved planta yield and also increased the mycelium length of AMF *Gigaspora rosea* by 100–200%, suggesting that *Epichloë* exudates enhance plant growth and yields, particularly through enhancing AMF development [70]. Furthermore, the association between *Bromus catharticus* and grass endophytes enhanced the mycorrhizal colonization of *Lolium multiflorum*, *Schedonorus arundinaceus*, and *Bromus catharticus* [71]. Colonization of *Lolium perenne* by mycorrhizal fungi reduced the abundance of grass endophytes and the secretion of anti-herbivore-associated alkaloids peramine and lolitrem B [46]. However, so far, the mechanism by which alkaloids produced by grass endophytes impact AMF development is not understood.

## 3. Effect of Concurrent Colonization of AMF and Grass Endophyte on Host Plant

### 3.1. Effect of Co-Colonization of AMF and Grass Endophyte on Host Plant Growth

The effects of AMF and grass endophyte interaction on plant growth are diverse, ranging from negative [54] to positive [55] and neutral [56] (Table 2). According to a greenhouse experiment, colonization with *Epichloë festucae* var. *lolii* and AMF *Claroideoglomus etunicatum* significantly increased the total biomass and dry weight of *Lolium perenne* [55]. Furthermore, concurrent colonization with the two fungi increased the plant’s P uptake and physiological indexes such as dry biomass, hormones, and root length. These findings suggest that the interaction between grass endophyte and AMF can positively affect plant growth. Liu et al. found a positive correlation between the shoot P concentrations and the abundance of AMF and grass endophyte [72]. Additionally, Wezowicz et al. reported that inoculating *Verbascum lychnitis* with both fungi significantly increased the abundance of Photosystem II protein and plant chlorophyll concentration [73].

Although grass endophytes and AMF can promote various growth parameters in plants, it should be noted that the beneficial effects of these two fungi are not always obvious. For example, simultaneous colonization with grass endophytes and AMF had no effect on the growth of *Bromus auleticus* [5]. Furthermore, *Neotyphodium occultans* (*Epichloë occultans*) and AMF did not improve the performance or nutrient content of *Lolium multiflorum* [74]. Surprisingly, when *Lolium arundinaceum* was infected with *Epichloë coenophialum*, inoculation with the AMF *Funneliformis mosseae* negatively impacted shoot and root biomass, while the presence of the AMF *Claroideoglomus etunicatum* significantly promoted plant growth [54]. This means that AM fungi can diminish the benefits of grass endophytes. These results indicate that in the tripartite interactions between grass endophytes, AMF, and host species, the benefits depend on the interactions between the AMF and host species, and the grass endophyte and AMF species. By gaining a deeper understanding of the mechanisms behind these effects, it may be possible to optimize the use of fungi to increase plant yield and improve quality.

**Table 2:** The effects of co-infection of arbuscular mycorrhizal fungi and grass endophyte on host plant.

Plants	Grass Endophyte	AM Fungi	Study Site	Results	References
*Lolium perenne*	*Epichloë festucae* var. *lolii*	*Claroideoglomus etunicatum*	Greenhouse	+44.53%, 30.27%, and 28.47% of dry weight in soil moisture conditions of 30%, 50%, and 70%.	[55]
*Leymus chinensis*	*Epichloë bromicola*	*Glomus etunicatum* *Glomus intraradices*	Greenhouse	+73.21% P absorption.	[48]
*Lolium arundinaceum*	*Epichloë coenophialum*	*Claroideoglomus etunicatum*; *Funneliformis mosseae*	Greenhouse	Inoculated with *F*. *mosseae* alone, −41.79% and −68.82% of shoot biomass and root biomass, had no impact in salt-alkali stress.	[54]
*Achnatherum sibiricum*	*Epichloë sibirica*	*Glomus mosseae*; *Glomus etunicatum*	Greenhouse	Greater competitive ability.	[4]
*Achnatherum sibiricum*	*Epichloë sibirica*	*Glomus mosseae*; *Glomus etunicatum*	Greenhouse	+12.5% and 10.55% of the total phenolic content when inoculated with GM and GE respectively.	[75]
*Lolium multiflorum*	*Neotyphodium occultans* (*Epichloë occultans*)	*Glomus mosseae*; *Glomus caledonium*; *Glomus fasciculatum*	Greenhouse	No impact.	[74]
*Lolium perenne*	*Epichloë typhina; Neotyphodium lolii* *(Epichloë festucae* var. *lolii*)	*Sclerocystis* sp.	Greenhouse	Higher shoot-root biomass ration.	[76]
*Lolium perenne*	*Epichloë festucae* var. *lolii*	*Claroideoglomus etunicatum*	Greenhouse	Highest values of SOD, POD, the total P content, and the total dry weight.	[43]

### 3.2. Effect of Simultaneous Colonization with AMF and Grass Endophyte on Biotic Resistance of Host Plant

Throughout the growth and development process, plants are subjected to different stresses from biotic and abiotic factors, and even experience both types of stress simultaneously. Previous studies have primarily focused on the effects of either grass endophytes or AMF on the host’s resistance to biotic or abiotic stress. Because AMF and grass endophytes simultaneously colonize the same host plant, it is essential to explore the combined effect of these fungi on the host plant’s resistance to stress. We believe that this interaction has the potential to significantly influence plant health and productivity. 

To date, a few researchers have demonstrated that AMF-grass endophyte-plant associations can enhance host plant tolerance to various stressors (Table 3). A greenhouse-based study demonstrated that inoculating ryegrass with both *Claroideoglomus etunicatum* and *Epichloë festucae* var. *lolii* reduced the severity of leaf spot caused by *B. sorokiniana*, and the levels of malondialdehyde and hydrogen peroxide were lower [43]. Similarly, a related study revealed a decrease in the abundance of pathogens due to the increased production of total phenols in *Achnatherum sibiricum* induced by the colonization of *Glomus etunicatum* and *Epichloë sibirica* [77].

Grass endophytes confer prominent advantages to host plants, such as alkaloid production, induction of expression of anti-herbivore feeding genes, and alteration of volatile substance composition, which discourages feeding by herbivores [78]. The enhancement of plant resistance by AMF is undeniable [25,79]. However, studies on the interactions between grass endophytes and AMF and the resulting repulsion of herbivores and other biological stresses by the host plant remain scanty.

At present, only a few studies have shown that the interactions between AMF and grass endophytes have reduced the feeding of some plants by herbivores. In 2002, Mark et al. used perennial ryegrass (*Lolium perenne* cv. “Express”) to investigate the effect of the interaction between *Glomus mosseae* and *Neotyphodium lolii* (*Epichloë festucae* var. *lolii*) on the noctuid *Phlogophora meticulosa* [80]. The study revealed that the presence of grass endophytes and mycorrhizal fungi cumulatively decreased the survival of second to fifth-instar larvae, and the effect was greater when the plant had adequate phosphorus. Additionally, there was a negative correlation between the interaction between the two fungi and feeding by insects. Notably, grass endophytes amplified the relative larval feeding rate while concurrently diminishing the insect’s food conversion efficiency. Conversely, mycorrhizal fungi had no obvious effect on the feeding behavior.

Pathogens and pest insects usually infect or feed on the same host plants. Currently, only a few studies have investigated the interplay between AMF and grass endophytes in relation to simultaneous infestation with pathogens and insects. Our understanding of the influence of plant defenses on symbiotic fungal endophytes is limited.

**Table 3:** Results of co-infection of arbuscular mycorrhizal fungi and grass endophyte on plant resistance to abiotic and biotic stress.

Plants	Grass Endophyte	AM Fungi	Stress	Results	References
*Lolium perenne*	*Epichloë festucae* var. *lolii*	*Claroideoglomus etunicatum*	Water	Enhanced uptake of phosphorus (P), elevated photosynthetic activity, and the accumulation of osmoregulatory compounds.	[55]
*Lolium arundinaceum*	*Epichloë* *coenophialum*	*Claroideoglomus etunicatum*; *Funneliformis mosseae*	Saline-alkali	CE significantly enhanced saline-alkali resistance by increasing potassium (K+) accumulation and reducing sodium (Na+) concentration, whereas resistance was reduced following inoculation with FM.	[54]
*Leymus chinensis*	*Epichloë bromicola*	*Funneliformis mosseae*	Drought	AMF enhanced the drought resistance of EF plants, yet had no significant effect on the drought resistance of EI plants.	[81]
*Lolium perenne* cv. “Express”	*Neotyphodium lolii* (*Epichloë festucae* var. *lolii*)	*Glomus mosseae*	Pest	Mycorrhizal and endophyte interaction was observed in third-instar larvae regarding survivorship.	[80]
*Lolium perenne*	*Epichloë festucae* var. *lolii*	*Claroideoglomus etunicatum*	Pathogen	Suppressed the occurrence of leaf spot by increasing the levels of chemical substances and the plant defensive enzymes.	[44]
*Lolium perenne*	*Epichloë festucae* var. *lolii*	*Claroideoglomus etunicatum*	Pathogen	Decrease disease incidence by 10.93%, elevated plant defensive activity levels but reduced concentrations of MDA and H_2_O_2_.	[43]
*Achnatherum sibiricum*	*Epichloë sibirica*	*Glomus etunicatum*	Pathogen	Increased production of total phenols of plants, and thus decreased the abundance of pathogen.	[77]

### 3.3. Effects of Simultaneous Colonization of AMF and Grass Endophyte on Host Plant Resistance to Abiotic Stress

Compared with research on biotic stress, research on the impact of simultaneous inoculation with AMF and grass endophytes on abiotic stressors, such as drought, saline-alkali, and water, is more well-established. Simultaneous colonization with AMF and grass endophytes was found to significantly increase the concentration of soluble sugar and peroxidase activity under 30% soil water content [55]. *Lolium arundinaceum* was inoculated with *Epichloë coenophialum* and either *Claroideoglomus etunicatum* or *Funneliformis mossea* under different saline-alkali contents. Interestingly, inoculation with *Epichloë coenophialum* and *Claroideoglomus etunicatum* increased the resistance of the host, compared to plants inoculated with the AMF *Funneliformis mossea* and *Epichloë coenophialum* [54]. Benefits of grass endophytes may be diminished or weakened by inoculation with *Funneliformis mossea*, and this has been reported in many studies. For instance, inoculating *Leymus chinensis* with *Epichloë bromicola* and *Funneliformis mosseae* weakened its drought stress tolerance [81].

Indeed, the effect of infection with two symbionts on plant resistance is a complex and multifaceted phenomenon, and the interaction between different factors needs to be further explored. While numerous studies have investigated the benefits and drawbacks of individual above-ground or below-ground microorganisms on host plants, only a few studies have investigated the tripartite interactions between grass endophytes, AM fungi, and host plants.

**Figure 1:**
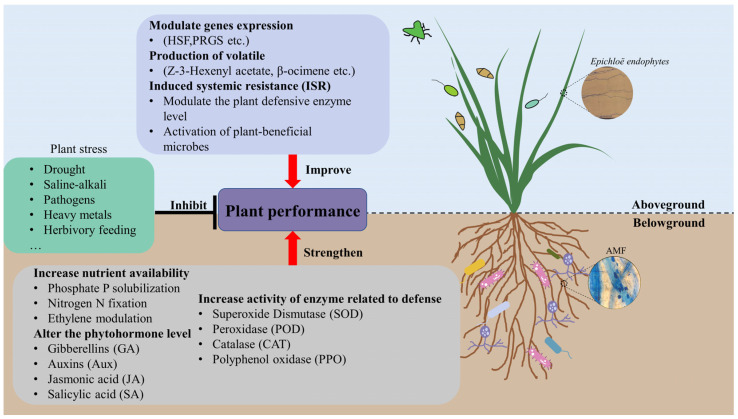
The potential mechanisms by which arbuscular mycorrhizal fungi (AMF) and grass endophyte (*Epichloë*) infection affect host plant response to biotic and abiotic stress.

Dual symbiotic fungal-plant interactions have been found to increase plant biomass, enhance the activity of defense enzymes, increase nutrient uptake rate, and modify net photosynthetic rate. For instance, a study conducted on *Lolium perenne* found that the grass endophyte *Epichloë* and AMF *Claroideoglomus etunicatum* increased the total dry weight and phosphorus (P) content of the plant and induced higher activity of defense enzymes, including peroxidase (POD), polyphenol oxidase (PPO), and catalase (CAT) and lower concentrations of malondialdehyde (MDA) under drought stress conditions [55]. The enhanced performance of host plants under drought stress could be attributed to the altered activity of anti-stress enzymes. In addition, co-infection with *Epichloë* and *Claroideoglomus etunicatum* enhanced the saline-alkali stress tolerance of *Lolium arundinaceum* by increasing K^+^ concentration and nutrient uptake while decreasing Na^+^ concentration [54]. These studies provide evidence that AMF and grass endophytes can simultaneously modulate the physiological responses of host plants to external stress (Figure 1).

Furthermore, the interaction between AMF and grass endophytes can alleviate stress in plants by modulating the response of secondary metabolites and their proportions and regulating the expression of genes related to stress. These factors can affect the growth and development of host plants, as well as their tolerance to stress. One research study demonstrated that co-inoculation with AMF *Claroideoglomus etunicatum* and grass endophyte *Epichloë festucae* var. *lolii* reduced the disease index caused by *B. sorokiniana* in ryegrass by increasing the accumulation of soluble protein [43]. Under certain conditions, combining AMF and grass endophytes may not always result in synergistic effects on the host plants. For instance, under water stress, mycorrhizal inoculation significantly increased proline content by 15% and total phenolic concentration by 18% in endophyte-free *Leymus chinensis*, but the treatment had no significant effects on endophyte-infected plants [81].

Only a few studies have investigated the mechanisms of interactions between AMF and grass endophytes and plants at the molecular level. Our research group conducted a study of AMF-grass endophyte-pathogen interactions in perennial ryegrass using RNA-seq analysis [82]. We found unexpected results that showed that both AMF and grass endophytes significantly increased the expression of genes related to the regulation of SOD, POD, PPO, and CAT activity. Interestingly, the number of genes whose expression was up-regulated induced by the interaction between AMF and grass endophytes was higher than infection with two fungi. Moreover, this interaction also induced terpenoid backbone biosynthesis, biotin metabolism, aldehyde metabolism, and the expression of proteins involved in plant-pathogen interactions. The up-regulation of SOD, PPO, POD, CAT, and SA-related genes also occurred. Furthermore, it also induced the expression of 11 pathogenesis-related genes (PRGs), and the Heat Shock Factor (HSF) significantly enhanced the disease resistance of plants (Figure 1). Future research should focus on this area to better understand events under multiple symbioses.

### 3.4. Effect of Simultaneous Colonization with AMF and Grass Endophyte on Plant Competitive Ability

Grass endophytes and AMF could positively impact the competitive ability of host plants [83,84,85]. Specifically, some studies have reported that grass endophytes mostly positively affect inter-specific or intra-specific competition and induce plant community shifts by promoting host growth, including increasing the number of tillers and enhancing root and shoot biomass or the production of allelopathic substances. However, only a few studies have investigated the effect of dual infections on the performance of plant competition. A study showed that the presence of both grass endophytes and AM fungi can enhance the growth performance and inter-specific competition ability of *Achnatherum sibiricum*, leading to changes in the plant community structure that promotes the coexistence of dominant species (*Stipa grandis*) and subordinate species (*Achnatherum sibiricum*) [4]. So far, the mechanisms underlying the effects of mycorrhizal inoculation and endophytes on altering plant community structure are poorly understood.

## 4. Conclusions and Future Perspectives

In recent years, plant-microbe interactions have gained considerable attention, and exploration of the interactions between plants and plant microbiomes is an important area for elucidating the mechanisms of interspecies interactions. Increasing evidence suggests that both above-ground and below-ground microbes provide various functions for their host plants, such as promoting growth, enhancing nutrient uptake, improving abiotic stress tolerance, and increasing biotic resistance and tolerance, including disease resistance [86,87,88,89,90]. In AM-*Epichloë*-plant associations, AMF and *Epichloë* endophyte increase nutrient uptake, enhance photosynthesis and increase phytohormone levels, enhance plant defense, alter the production of volatile compounds, modulate plant resistance related gene expression, and increase plant resistance to biotic and abiotic stress (Figure 1). The interactions between above-ground and below-ground microorganisms and their impact on plants have long been recognized as a crucial frontier for comprehending fundamental biochemical and ecological processes in both agricultural and natural ecosystems. In addition, the current limitations of research methods and data analysis models have impeded our understanding of these interactions and have hindered our ability to effectively leverage microbial resources to enhance productivity and ecological value. Herein, we highlighted the interactions between above-ground microorganisms (grass endophytes) and below-ground microorganisms (AMF) and how multiple symbionts shape the performance of host plants under different stress conditions. We propose that the species, and whether they occur alone or simultaneously with other symbiotic fungi, are the most important factors that determine whether they confer beneficial, harmful, or neutral effects to the host plants. The effects of grass endophytes and AMF on host plant growth and development depend on the degree of host signals and environmental stress. Simultaneous infection with fungi plays a critical role in determining host plant performance, and the relative importance and functional effects of these symbiotic processes vary depending on plant species, developmental stage, and microbial community.

Despite recent advances significantly expanding our understanding of the symbiotic processes and functions of grass endophytes and AM fungi, our knowledge of the molecular mechanisms underlying simultaneous infections and their effect on plant defenses and growth remains limited. Moreover, the practical applications of multiple plant-microbe interactions in sustainable agriculture and the protection of ecology still lag behind. For example, some critical research areas remain largely unexplored: (1) How do *Epichloë*-colonized plants recruit AMF at different growth stages? (2) Which kinds of keystone functional genes in the *Epichloë*-AMF-plant system enhance host resistance to biotic and abiotic stresses? (3) Can, and how do, the *Epichloë*-AMF-plant systems recruit beneficial microbes with desired functions under various environmental conditions? (4) How do *Epichloë*-AMF-plant complexes and their microbiomes interact and co-evolve in response to different agricultural management practices and global climatic changes over a long time? (5) How do grass endophytes and AMF affect the movement and utilization of nutrients in the above-ground and below-ground plant parts? (6) What is the function and role of AMF-*Epichloë*-plant symbionts on carbon fixation, species diversity, community stability, and the productivity of the ecosystem? Answering these questions can contribute to our knowledge of the underlying mechanisms of *Epichloë*-AMF-plants interactions and provide essential information for precisely harnessing beneficial microbiomes, including developing agricultural sustainability.

## Data Availability

All applicable data are published and referenced in the paper.
